# Prevalence and Antimicrobial Susceptibility of Mycoplasma hominis and *Ureaplasma* Species in Nonpregnant Female Patients in South Korea Indicate an Increasing Trend of Pristinamycin-Resistant Isolates

**DOI:** 10.1128/AAC.01065-20

**Published:** 2020-09-21

**Authors:** Ji Yong Lee, Jeong Seon Yang

**Affiliations:** a Division of Infectious Diseases, Department of Internal Medicine, H Plus Yangji Hospital, Seoul, South Korea; b Department of Laboratory Medicine, H Plus Yangji Hospital, Seoul, South Korea

**Keywords:** *Mycoplasma hominis*, *Ureaplasma* species, antimicrobial susceptibility, prevalence, pristinamycin

## Abstract

Mycoplasma hominis and *Ureaplasma* species, commonly found in the lower urogenital tract, have been associated with various urogenital infections. This study aimed to estimate the prevalence and antimicrobial susceptibility trend of M. hominis and *Ureaplasma* sp. in female patients and to evaluate the risk factors for the acquisition of pristinamycin-resistant mycoplasma. Endocervical swab specimens obtained between March 2016 and December 2018 were analyzed using a Mycoplasma IST2 kit. Because pristinamycin and josamycin are not available in South Korea, we conducted an age- and date-matched case-control study to evaluate the risk factors for the acquisition of pristinamycin-resistant isolates. Among 4,035 specimens, 1,589 (39.4%) cases were positive for genital mycoplasma, which included 49 (3.1%) cases of M. hominis, 1,243 (78.2%) cases of *Ureaplasma* sp., and 297 (18.7%) cases of both M. hominis and *Ureaplasma* species. Based on antimicrobial susceptibility tests, the antibiotic susceptible rate of both M. hominis and *Ureaplasma* species to pristinamycin decreased annually during the study period (100%, 97.1%, and 87.3% for 2016, 2017, and 2018, respectively, *P* < 0.001). According to a multivariate analysis, josamycin resistance (odds ratio, 7.18; 95% confidence interval, 1.20 to 43.00; *P* = 0.027) and coinfection (odds ratio, 145.38; 95% confidence interval, 21.80 to 3,017.23; *P* < 0.001) with *Candida* species were independent risk factors for the acquisition of pristinamycin-resistant isolates. Antibiotic-resistant genital mycoplasmas have been gradually increasing annually. Nationwide surveillance, proper antibiotic stewardship, and appropriate culture-based treatment strategies are required to control this upcoming threat.

## Abstract (precis)

Mycoplasma hominis and *Ureaplasma* species, commonly found in the lower urogenital tract, have been associated with various urogenital infections. This study aimed to estimate the prevalence and antimicrobial susceptibility trend of M. hominis and *Ureaplasma* sp. in female patients and to evaluate the risk factors for the acquisition of pristinamycin-resistant mycoplasma. Endocervical swab specimens obtained between March 2016 and December 2018 were analyzed using a Mycoplasma IST2 kit.

## INTRODUCTION

Mycoplasma hominis and *Ureaplasma* species, including Ureaplasma parvum and Ureaplasma urealyticum, are facultative anaerobic organisms that are commonly found in the lower urogenital tract. These organisms are considered etiologic agents causing various urogenital diseases in women, such as cervicitis, cystitis, bacterial vaginosis, pelvic inflammatory disease, chorioamnionitis, postpartum fever, infertility, prematurity, intrauterine growth retardation, and systemic neonatal infections (1).

Genital mycoplasmas are not susceptible to beta-lactam antibiotics and glycopeptides because of the absence of the cell walls of these mycoplasmas. Historically, *Ureaplasma* species has been found to be susceptible to the tetracycline and macrolide classes of antibiotics (2). M. hominis is uniformly resistant to the macrolide drugs currently available but is generally susceptible to tetracyclines. However, some studies have reported that the incidence of tetracycline resistance associated with acquisition of the *tet*(M) determinant has been increasing (1, 3, 4).

The prevalence and antibiotic susceptibility profiles vary geographically and depend on the use of different antibiotics and the history of previous antibiotic exposure (5, 6). It is important to identify the prevalence and antibiotic susceptibility profiles of genital mycoplasmas so that sufficient information is available when selecting appropriate empirical antibiotics and when performing antibiotic stewardship. This study aimed to investigate the prevalence and antibiotic resistance profiles of M. hominis and *Ureaplasma* species isolates from nonpregnant female patients in South Korea.

## RESULTS

### Detection of genital mycoplasmas.

Out of the 4,035 samples, the prevalence of genital mycoplasma infection was 39.4% (1,589 of 4,035). Of the 1,589 samples with a positive culture, 49 (1.2%) had M. hominis only, 1,243 (30.8%) had *Ureaplasma* species only, and 297 (7.4%) had both M. hominis and *Ureaplasma* species. Thus, *Ureaplasma* species infection was significantly more prevalent than M. hominis infection. The results for the distribution of M. hominis and *Ureaplasma* species, according to age group, are presented in Table 1. Genital mycoplasma infections were more prevalent in younger patients (cutoff value, 56; *P* < 0.001).

**TABLE 1:** Distribution of Mycoplasma hominis and *Ureaplasma* species infection in different age groups of female patients

Age (yr)	Distribution [no. (%)]				Overall no.
	M. hominis (*n* = 49)	*Ureaplasma* species (*n* = 1,243)	Both^*a*^ (*n* = 297)	Positive (*n* = 1,589)	
18–29	5 (1.0)	167 (34.9)	38 (7.9)	210 (43.8)	479
30–39	10 (1.5)	227 (35.0)	36 (5.5)	273 (42.1)	649
40–49	14 (1.2)	381 (33.9)	101 (9.0)	496 (44.1)	1,124
50–59	11 (0.9)	365 (29.7)	89 (7.2)	465 (37.8)	1,129
60–89	9 (1.6)	103 (18.6)	33 (6.0)	145 (26.2)	554

a Both, infected by Mycoplasma hominis and *Ureaplasma* species.

**FIG 1:**
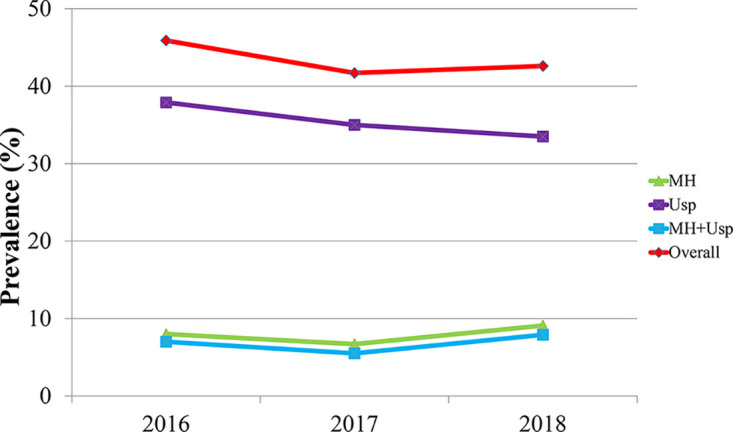
Trends in the prevalence of Mycoplasma hominis and *Ureaplasma* species between 2016 and 2018. MH, Mycoplasma hominis; Usp, *Ureaplasma* species; MH+Usp, infected by Mycoplasma hominis and *Ureaplasma* species simultaneously.

Trends in the prevalence of genital mycoplasmas during the 3-year period between 2016 and 2018 are shown in Fig. 1. The prevalence of *Ureaplasma* species infection significantly decreased during the analyzed period (*P* = 0.04). Although there was no significant difference, the prevalence of M. hominis infection increased during that period (*P* = 0.08).

### Antimicrobial susceptibility patterns.

The analysis of the antimicrobial susceptibility patterns of M. hominis, *Ureaplasma* species, and both M. hominis and *Ureaplasma* species in the 1,589 culture-positive samples showed that most isolates were not fully susceptible to all nine antibiotics (Table 2). Only M. hominis isolates were fully susceptible to pristinamycin. More than 60% of M. hominis isolates were susceptible to ofloxacin and ciprofloxacin, whereas only 6% and 18.7% of *Ureaplasma* species isolates were susceptible to these respective quinolones. A total of 72.3% of M. hominis isolates and 90.7% of *Ureaplasma* species isolates were susceptible to tetracycline, whereas more than 93% of genital mycoplasma isolates were susceptible to doxycycline. More than 85% of *Ureaplasma* species isolates were susceptible to erythromycin, clarithromycin, and azithromycin (85.8%, 98.0%, and 85.0%, respectively). More than 99% of M. hominis and *Ureaplasma* species isolates were susceptible to josamycin and pristinamycin, respectively. However, about 5% of both M. hominis and *Ureaplasma* species groups were resistant to josamycin and pristinamycin.

**TABLE 2:** Antimicrobial susceptibilities of Mycoplasma hominis and *Ureaplasma* species for all patients^*a*^ (*n* = 1,589)

Antimicrobial and sensitivity level	Susceptibility [no. (%)] for:		
	M. hominis (*n* = 49)	*Ureaplasma* species (*n* = 1,243)	Both^*a*^ (*n* = 297)
Ciprofloxacin			
S	67 (67.3)	75 (6.0)	16 (5.4)
I	9 (18.4)	424 (34.1)	64 (21.5)
R	7 (14.3)	744 (59.9)	217 (73.1)
Ofloxacin			
S	34 (69.4)	233 (18.7)	27 (9.1)
I	7 (14.3)	676 (54.4)	143 (48.1)
R	8 (16.3)	334 (26.9)	127 (42.8)
Tetracycline			
S	35 (71.4)	1,120 (90.1)	215 (72.4)
I	3 (6.1)	42 (3.4)	29 (9.8)
R	11 (22.4)	81 (6.5)	53 (17.8)
Doxycycline			
S	48 (98.0)	1,169 (94.0)	276 (92.9)
I	1 (2.0)	26 (2.1)	12 (4.0)
R	0 (0)	48 (3.9)	9 (3.0)
Erythromycin			
S	NR	1,066 (85.8)	6 (2.0)
I	NR	165 (13.3)	33 (11.1)
R	NR	12 (1.0)	258 (86.9)
Clarithromycin			
S	NR	1,218 (98.0)	21 (7.1)
I	NR	14 (1.1)	25 (8.4)
R	NR	11 (0.9)	251 (84.5)
Azithromycin			
S	NR	1,056 (85.0)	9 (3.0)
I	NR	183 (14.7)	66 (22.2)
R	NR	4 (0.3)	222 (74.7)
Josamycin			
S	47 (95.9)	1,235 (99.4)	253 (85.2)
I	2 (4.1)	7 (0.6)	29 (9.8)
R	0 (0)	1 (0.1)	15 (5.1)
Pristinamycin			
S	49 (100)	1,239 (99.7)	280 (94.3)
R	0 (0)	4 (0.3)	17 (5.7)

a Both, infected by Mycoplasma hominis and *Ureaplasma* species; S, susceptible; I, intermediate; R, resistant; NR, natural resistance.

**FIG 2:**
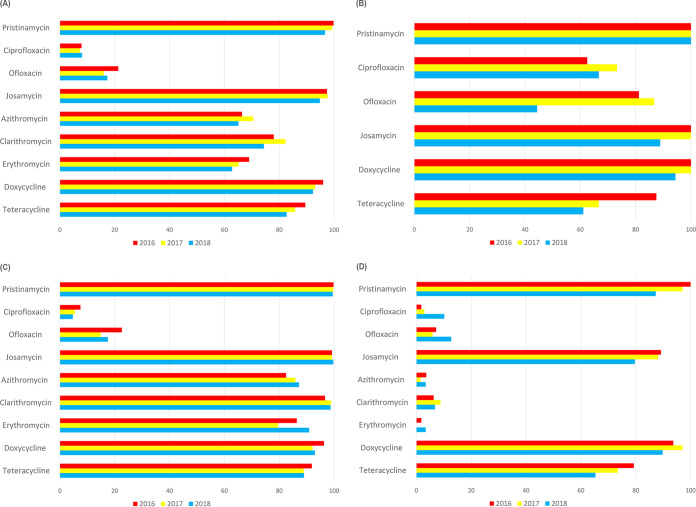
Antimicrobial susceptibility patterns of Mycoplasma hominis and *Ureaplasma* species during 2016 to 2018. (A to D) Antibiotic susceptibility patterns of overall genital mycoplasmas (A), Mycoplasma hominis (B), *Ureaplasma* species (C), and both Mycoplasma hominis and *Ureaplasma* species (D).

The susceptibility rates of M. hominis and *Ureaplasma* species did not significantly change during the study period, except for susceptibility rates of *Ureaplasma* species to erythromycin. However, the susceptibility rates of both M. hominis and *Ureaplasma* species to pristinamycin decreased annually (100%, 97.1%, and 87.3%, respectively; *P* < 0.001). The susceptibility rates of M. hominis, *Ureaplasma* species, and both M. hominis and *Ureaplasma* species are described in Fig. 2.

### Risk factor analysis for infection by pristinamycin-resistant genital mycoplasmas.

The baseline characteristics of patients who were infected with pristinamycin-resistant genital mycoplasmas are presented in Table 3. No patient was infected by Trichomonas vaginalis in either group. In the univariate analysis using a logistic regression model, resistance to erythromycin, josamycin, and tetracycline, infection by *Ureaplasma* species, and coinfection with *Candida* species were risk factors for pristinamycin-resistant mycoplasma infection. In the multivariate analysis, josamycin resistance and coinfection with *Candida* species were considered independent risk factors (Table 4).

**TABLE 3:** Baseline characteristics of 21 patients of the case group and 53 patients of the control group, including antibiotic susceptibility profiles

Parameter^*f*^	Value(s) [no. (%)] for:		*P* value
	Case (*n* = 21)	Control (*n* = 53)	
Date			0.917
2016	1 (4.8)	2 (3.8)	
2017	3 (14.3)	6 (11.3)	
2018	17 (81.0)	45 (84.9)	
Age, yr [median (IQR)]	51.0 (34.0; 57.0)	46.0 (31.0; 55.0)	0.529
Detected mycoplasma			0.014
M. hominis	0 (0.0)	4 (7.5)	
*Ureaplasma* species	4 (19.0)	26 (49.1)	
Both^*a*^	17 (81.0)	23 (43.4)	
Specific symptom^*b*^	18 (85.7)	47 (88.7)	0.707
Previous antibiotics^*c*^	6 (28.6)	11 (20.8)	0.544
Recurrent infection^*d*^	3 (14.3)	15 (28.3)	0.334
IUD state	1 (4.8)	2 (3.8)	1.000
Coinfection by *Candida* species^*e*^	5 (23.8)	5 (9.4)	0.135
Antibiotics susceptibility			
Tetracycline	11 (52.4)	42 (79.2)	0.043
Doxycycline	21 (100.0)	49 (92.5)	0.572
Ofloxacin	17 (81.0)	38 (71.7)	0.599
Ciprofloxacin	13 (61.9)	23 (43.4)	0.239
Erythromycin	5 (23.8)	30 (56.6)	0.022
Clarithromycin	6 (28.6)	29 (54.7)	0.076
Azithromycin	9 (42.9)	31 (58.5)	0.338
Josamycin	7 (33.3)	52 (98.1)	<0.001

a Infected by both of Mycoplasma hominis and *Ureaplasma* species.

b Abnormal discharge, dysuria, or other voiding symptoms, dyspareunia, bleeding between periods or after sex, fever, and low abdominal pain.

c History of any antibiotic administration for any reasons within 3 months.

d History of infection by M. hominis, *Ureaplasma* species, or both M. hominis and *Ureaplasma* species within 3 months before exam.

e Confirmed by wet smear test.

f IQR, interquartile range; IUD, intrauterine device.

**TABLE 4:** Risk factors associated with infection by pristinamycin-resistant Mycoplasma hominis and *Ureaplasma* species

Factor	Univariate analysis		Multivariate analysis	
	Odds ratio (95% CI^*f*^)	*P* value	Odds ratio (95% CI)	*P* value
*Ureaplasma* species	0.21 (0.05–0.66)	0.012		
Specific symptom^*a*^	0.77 (0.18–3.93)	0.726		
Previous antibiotics^*b*^	1.53 (0.46–4.79)	0.473		
Coinfection by Candida species^*c*^	3.00 (0.75–12.14)	0.114	7.18 (1.20–43.00)	0.027
Recurrent infection^*d*^	0.42 (0.09–1.48)	0.214		
Resistance^*e*^				
JOS	104.00 (17.16–2040.23)	<0.001	145.38 (21.80–3017.23)	<0.001
ERY	4.17 (1.41–14.31)	0.008		
CLA	3.02 (1.05–9.59)	0.047		
AZT	1.88 (0.68–5.35)	0.227		
TET	3.47 (1.18–10.48)	0.024		
OFX	0.60 (0.15–1.94)	0.600		
CIP	0.47 (0.16–1.31)	0.155		

a Abnormal discharge, dysuria, or other voiding symptoms, dyspareunia, bleeding between periods or after sex, fever, and low abdominal pain.

b History of any antibiotic administration for any reasons within 3 months.

c Confirmed by wet smear test.

d History of infection by M. hominis, Usp, or both M. hominis and Usp within 3 months before exam.

e Resistance to each antibiotic: JOS, josamycin; ERY, erythromycin; CLA, clarithromycin; AZT, azithromycin; TET, tetracycline; OFX, ofloxacin; CIP, ciprofloxacin.

f CI, confidence interval.

## DISCUSSION

In this study, the prevalence of genital mycoplasma was 39.4% in symptomatic female patients. The prevalence of genital mycoplasmas did not show a significant decrease during the study period. Although direct comparison is difficult because there is no existing study assessing the trend of prevalence of genital mycoplasmas in symptomatic female patients in South Korea, Lee et al. reported that there was no significant difference in the prevalence of overall genital mycoplasmas between 2009 and 2013 every year in pregnant women (7). The prevalence of *Ureaplasma* species did not change significantly over the study period according to Lee et al.’s study, but it significantly decreased in our study. Interestingly, the prevalence of M. hominis and mixed infection increased significantly in both studies.

The prevalence of *Ureaplasma* species (30.8%) was higher than that of M. hominis (1.2%). According to several studies conducted in South Korea, the prevalence of *Ureaplasma* species in symptomatic patients was higher than that of M. hominis. The prevalence of *Ureaplasma* species and M. hominis was 21.3% and 2.9%, as reported by Moon et al. (8), 65.6% and 11.8% by Kweon et al. (9), and 48.8% and 25.3% by Jang et al. (10), respectively. Similar values were reported in Poland (11) and China (12).

Regarding antimicrobial susceptibility, the majority of genital mycoplasmas were most susceptible to pristinamycin, followed by josamycin, doxycycline, and tetracycline, but most of them were resistant to ciprofloxacin and ofloxacin. The majority of M. hominis isolates were resistant to erythromycin, clarithromycin, and azithromycin, whereas more than 85% of *Ureaplasma* species isolates were susceptible to these antibiotics. M. hominis is intrinsically resistant to C14- and C15-membered macrolides, for example, erythromycin, clarithromycin, and azithromycin, but susceptible to C16-membered macrolides, for example, josamycin. Pereyre et al. reported that intrinsic erythromycin resistance of M. hominis was linked to the G2057A transition in domain V of the 23S rRNA sequence, and the high-level macrolide resistance might be associated with additional C2610U transition or the presence of a putative efflux mechanism (13). They also described that two josamycin-resistant isolates of M. hominis contained A2059G and C2611U mutations.

Resistance to pristinamycin (these drugs are not available for clinical prescription in South Korea) was not frequently observed among both *Ureaplasma* species and M. hominis isolates but showed an upward trend during the study period. Resistance to doxycycline also showed an upward trend among *Ureaplasma* species isolates. Studies assessing the resistant trend for antibiotics in South Korea have not been conducted yet. Kasprzykowska et al. reported that there was no isolate with resistance for pristinamycin or josamycin among genital mycoplasmas between 2003 and 2015 (11). A study from Hungary reported that 10% of *Ureaplasma* species were resistant to josamycin from the specimen collected from men and women during 2 years from 2008 (14). However, they used another antibiotic susceptibility test kit. There are some *in vitro* studies investigating macrolide resistance in M. hominis (15) and Ureaplasma parvum (16) by the same team. They selected macrolide-resistant mutants of M. hominis and *U. parvum* by serial passages of M. hominis isolates in medium containing subinhibitory concentrations of macrolides, lincosamides, streptogramins, and ketolides. Selection of the pristinamycin-resistant M. hominis strains was performed with clindamycin, pristinamycin, quinopristin-dalfopristin, telithromycin, and josamycin. Selection of the pristinamycin-resistant *U. parvum* strains also was performed with erythromycin, josamycin, quinopristin/dalfopristin, and telithromycin.

In our age- and date-matched case-control study, resistance to josamycin and coinfection with *Candida* species were considered independent clinical risk factors for pristinamycin-resistant mycoplasma infection.

This study has some limitations. First, the Mycoplasma IST2 kit test was unable to differentiate *U. urealyticum* from *U. parvum. U. parvum* differs from *U. urealyticum* in its antimicrobial susceptibility; thus, inability to differentiate these species in a sample may lead to inappropriate reporting of antibiotic susceptibility (5). Second, antimicrobial susceptibility test using the Mycoplasma IST2 kit may not be compatible with the standardized guidelines, such as those of the Clinical and Laboratory Standards Institute or the European Committee on Antimicrobial Susceptibility Testing (5, 11). Third, we could not conduct laboratory studies to find the mechanism of pristinamycin resistance of *Ureaplasma* species and M. hominis, because the specimens were discarded after they were tested with Mycoplasma IST2 kits. Fourth, there could be some selection bias in our case-control study.

### Conclusions.

The results of this study provide important epidemiological data concerning the prevalence and antimicrobial susceptibility patterns of *Ureaplasma* species and M. hominis over the recent 3-year period. The analysis showed that (i) approximately 40% of symptomatic female patients may have genital mycoplasma infection, (ii) doxycycline and tetracycline are good treatment options, and (iii) antimicrobial susceptibility tests for patients showing genital candidiasis should be considered. More comprehensive and large-scale surveillance studies with standardized methodologies are required, especially when assessing the resistant trend of new macrolides.

## MATERIALS AND METHODS

This study was conducted at H Plus Yangji Hospital, a 350-bed general hospital in Seoul, South Korea. This hospital performs genital mycoplasma culture, including antimicrobial susceptibility tests, with an endocervical or vaginal swab for patients who have specific symptoms or signs of genital tract infection or have abnormal Pap smear results during health screening. Clinical and microbiological data of female patients were collected from the database between 1 May 2016 and 31 December 2018. Patients older than 18 years were included in the study. Considering the large number of cases (more than 3,000 tests were performed annually) in this study, we selected the data of patients who were tested from May to July and November to December for each year. The following data were collected: age, results of genital mycoplasma tests, including the presence of M. hominis or *Ureaplasma* species, and results of antimicrobial susceptibility tests.

We also conducted a 1:2 age- and date-matched case-control study to identify the clinical risk factors for the acquisition of pristinamycin-resistant M. hominis and *Ureaplasma* species. Medical records were reviewed for the case and control groups. The following data were collected: age, sex, presence of specific symptoms, history of previous antibiotic administration within 3 months before examination, history of infection by M. hominis and *Ureaplasma* species within 3 months before examination, and presence of coinfection with *Candida* species or Trichomonas vaginalis. The specific symptoms were considered present when the patient described having abnormal discharge, dysuria, or other voiding symptoms, dyspareunia, bleeding between menstrual periods or after sexual intercourse, fever, and low abdominal pain (11). This study was approved by the Institutional Review Board of the H Plus Yangji Hospital.

A Mycoplasma IST2 kit (bioMérieux, Marcy-l’Etoile, France) was used for the detection, enumeration, identification, and antibiotic susceptibility testing for M. hominis and *Ureaplasma* species. Clinical specimens were inoculated in liquid transport medium R1 containing selective agents to inhibit the growth of contaminating flora in the sample. The samples in the R1 transport medium were centrifuged for 10 s and used to rehydrate the lyophilized selective growth medium R2. This medium was subsequently dispensed into 22 test wells, each well with a depth of 55 μl, and two drops of mineral oil were overlaid on each compartment to prevent desiccation. The strips were incubated at 37°C for 48 h and observed for color changes. Positive results were observed when the color of the culture medium changed from yellow to red due to alkalization and when the estimated density of each organism was ≥10^4^ CFU.

There were two concentration assay wells for each of the nine antibiotics (doxycycline, josamycin, ofloxacin, erythromycin, tetracycline, ciprofloxacin, azithromycin, clarithromycin, and pristinamycin). The development or absence of red color on the strip provided an index of the resistance or susceptibility, respectively, to each antimicrobial agent. The absence of red discoloration in either of the wells implied *Mycoplasma* sensitivity, whereas the presence of red discoloration in both wells signified *Mycoplasma* resistance. *Mycoplasma* was considered moderately susceptible to the antibiotic tested if the low-concentration assay wells turned red. The breakpoints for the antimicrobials tested were the following: tetracycline susceptible (S), ≤4; resistant (R), ≥8; doxycycline S, ≤4; R, ≥8; azithromycin S, ≤0.12; R, ≥4; clarithromycin S, ≤1; R, ≥4; erythromycin S, ≤1; R ≥4; josamycin S, ≤2; R, ≥8; ciprofloxacin S, ≤1; R, ≥2; ofloxacin S, ≤1; R, ≥4; and pristinamycin S, ≤1; R, ≥2 (17).

### Statistical analysis.

Student's *t* test and the Mann-Whitney U test were used to compare continuous variables, and the chi-square test and Fisher’s exact test were used for comparison of categorical variables. *P *values of <0.05 were considered statistically significant. To identify risk factors for the acquisition of pristinamycin-resistant M. hominis and *Ureaplasma* species, a logistic regression model was used to control for confounding variables. All *P* values were two-tailed. Variables that were statistically significant (*P* < 0.2) in the univariate analyses were included as candidates for multivariate analysis, in addition to the main variable of interest. The final logistic regression model was selected by stepwise backward elimination. Statistical analyses were performed using R, version 3.4.4.

## References

[1] Waites KB, Katz B, Schelonka RL 2005 Mycoplasmas and ureaplasmas as neonatal pathogens. Clin Microbiol Rev 18:757–789. doi:10.1128/CMR.18.4.757-789.2005. 16223956 PMC1265909

[2] Stimson JB, Hale J, Bowie WR, Holmes KK 1981 Tetracycline-resistant Ureaplasma urealyticum: a cause of persistent nongonococcal urethritis. Ann Intern Med 94:192–194. doi:10.7326/0003-4819-94-2-192. 7469210

[3] Degrange S, Renaudin H, Charron A, Bebear C, Bebear CM 2008 Tetracycline resistance in Ureaplasma spp. and Mycoplasma hominis: prevalence in Bordeaux, France, from 1999 to 2002 and description of two tet(M)-positive isolates of M. hominis susceptible to tetracyclines. Antimicrob Agents Chemother 52:742–744. doi:10.1128/AAC.00960-07. 18025113 PMC2224736

[4] Mardassi BB, Aissani N, Moalla I, Dhahri D, Dridi A, Mlik B 2012 Evidence for the predominance of a single tet(M) gene sequence type in tetracycline-resistant Ureaplasma parvum and Mycoplasma hominis isolates from Tunisian patients. J Med Microbiol 61:1254–1261. doi:10.1099/jmm.0.044016-0. 22580915

[5] Beeton ML, Spiller OB 2017 Antibiotic resistance among Ureaplasma spp. isolates: cause for concern? J Antimicrob Chemother 72:330–337. doi:10.1093/jac/dkw425. 27798207

[6] Choi JB, Lee SJ, Lee MK, Lee SJ, Park DC, Kim HY, Lee DS, Choe HS 2018 Prevalence and antimicrobial susceptibility of Ureaplasma spp. and Mycoplasma hominis in asymptomatic individuals in Korea. Microb Drug Resist 24:1391–1396. doi:10.1089/mdr.2017.0431. 29708840

[7] Lee MY, Kim MH, Lee WI, Kang SY, Jeon YL 2016 Prevalence and antibiotic susceptibility of Mycoplasma hominis and Ureaplasma urealyticum in pregnant women. Yonsei Med J 57:1271–1275. doi:10.3349/ymj.2016.57.5.1271. 27401661 PMC4960396

[8] Moon SJ, Choi J-E, Park K-I 2013 Comparison of the Anyplex II STI-7 and Seeplex STD6 ACE detection kits for the detection of sexually transmitted infections. J Lab Med Qual Assur 35:87–92. http://www.jlmqa.org/journal/view.html?volume=35&number=2&spage=87&vmd=Full.

[9] Kweon OJ, Lim YK, Oh SM, Kim T-H, Choe H-S, Lee S-J, Cho Y-H, Lee M-K 2016 Prevalence and antimicrobial susceptibility of Mycoplasma hominis, Ureaplasma urealyticum and Ureaplasma parvum in individuals with or without symptoms of genitourinary infections. Lab Med Online 6:79. doi:10.3343/lmo.2016.6.2.79.

[10] Jang YS, Min JW, Kim YS 2019 Positive culture rate and antimicrobial susceptibilities of Mycoplasma hominis and Ureaplasma urealyticum. Obstet Gynecol Sci 62:127–133. doi:10.5468/ogs.2019.62.2.127. 30918881 PMC6422850

[11] Kasprzykowska U, Sobieszczańska B, Duda-Madej A, Secewicz A, Nowicka J, Gościniak G 2018 A twelve-year retrospective analysis of prevalence and antimicrobial susceptibility patterns of Ureaplasma spp. and Mycoplasma hominis in the province of Lower Silesia in Poland. Eur J Obstet Gynecol Reprod Biol 220:44–49. doi:10.1016/j.ejogrb.2017.11.010. 29154180

[12] Wang QY, Li RH, Zheng LQ, Shang XH 2016 Prevalence and antimicrobial susceptibility of Ureaplasma urealyticum and Mycoplasma hominis in female outpatients, 2009–2013. J Microbiol Immunol Infect 49:359–362. doi:10.1016/j.jmii.2014.06.007. 25081985

[13] Pereyre S, Gonzalez P, De Barbeyrac B, Darnige A, Renaudin H, Charron A, Raherison S, Bébéar C, Bébéar CM 2002 Mutations in 23S rRNA account for intrinsic resistance to macrolides in Mycoplasma hominis and Mycoplasma fermentans and for acquired resistance to macrolides in M. hominis. Antimicrob Agents Chemother 46:3142–3150. doi:10.1128/aac.46.10.3142-3150.2002. 12234836 PMC128781

[14] Farkas B, Ostorházi E, Tóth B, Adlan E, Párducz L, Marschalkó M, Kárpáti S, Rozgonyi F 2011 Frequency and antibiotic resistance of Ureaplasma urealyticum and Mycoplasma hominis in genital samples of sexually active individuals. Orv Hetil 152:1698–1702. doi:10.1556/OH.2011.29217. 21979223

[15] Pereyre S, Renaudin H, Charron A, Bébéar C, Bébéar CM 2006 Emergence of a 23S rRNA mutation in Mycoplasma hominis associated with a loss of the intrinsic resistance to erythromycin and azithromycin. J Antimicrob Chemother 57:753–756. doi:10.1093/jac/dkl026. 16464889

[16] Pereyre S, Métifiot M, Cazanave C, Renaudin H, Charron A, Bébéar C, Bébéar CM 2007 Characterisation of in vitro-selected mutants of Ureaplasma parvum resistant to macrolides and related antibiotics. Int J Antimicrob Agents 29:207–211. doi:10.1016/j.ijantimicag.2006.09.008. 17196370

[17] De Francesco MA, Caracciolo S, Bonfanti C, Manca N 2013 Incidence and antibiotic susceptibility of Mycoplasma hominis and Ureaplasma urealyticum isolated in Brescia, Italy, over 7 years. J Infect Chemother 19:621–627. doi:10.1007/s10156-012-0527-z. 23192735

